# Acute 17β-Estradiol Administration Enhances Fear Extinction Memory and Alters Gut Microbiota in Female Rats

**DOI:** 10.1016/j.bpsgos.2025.100620

**Published:** 2025-09-26

**Authors:** Cassandra E. Hartsgrove, Genelle-Marie S. Walker, Karina D. Silva, Kailee Nunez, Karina Alejos, Makayla Joseph, Justin R. Wright, Brittney McMullen, Regina Lamendella, Lisa Y. Maeng

**Affiliations:** a Department of Psychology, Developmental and Brain Sciences Program, University of Massachusetts Boston, Boston, Massachusetts; b Department of Psychology, University of Massachusetts Boston, Boston, Massachusetts; c Honors College, University of Massachusetts Boston, Boston, Massachusetts; d Wright Labs, LLC, Huntington, Pennsylvania

**Keywords:** Anxiety, Estradiol, Estrobolome, Extinction memory, Fear conditioning, Fear extinction, Gut microbiome

## Abstract

**Background:**

Previous studies have shown that fear extinction memory and the gut microbiome are impacted by ovarian hormones. Elevated 17β-estradiol (E2) levels during fear extinction learning have been shown to enhance fear extinction recall 24 hours later. In this study, we concurrently examined the long-term maintenance of this facilitation and the role of the gut microbiome as a potential mediator.

**Methods:**

Naturally cycling adult female Sprague Dawley rats underwent an auditory-cued fear conditioning/extinction paradigm, during which the estrous cycle was tracked and fecal samples were collected. Habituation and conditioning took place when the rats were in estrus on day 1. On day 2, rats were administered either a sesame oil vehicle (*n* = 24) or E2 (15 μg/kg) (*n* = 25) before extinction training. Recent recall took place 24 hours after extinction training, and remote recall took place 1 to 2 weeks after extinction training (vehicle *n* = 13, E2 *n* = 12).

**Results:**

E2-treated rats showed significantly lower freezing behavior compared with vehicle-treated rats during recent recall, but not remote recall. Gut bacterial analysis using 16S ribosomal RNA sequencing revealed unique enrichment of estrogen-regulating and anxiety-related bacterial families during remote recall.

**Conclusions:**

The current data suggest that E2-enhanced fear extinction consolidation may be linked to alterations in gut microbiome composition. These findings may reveal a novel potential target for anxiety and other fear-based psychiatric disorders.

## Plain Language Summary

Estradiol influences gut bacteria composition and can also improve fear extinction, the process of learning to reduce fear. Higher estradiol levels can strengthen extinction memory, but the underlying mechanisms remain unclear. In this study, female rats received estradiol before fear extinction training. Rats that received estradiol showed less fear the day after training, but this effect did not last over the following weeks. Estradiol treatment also changed certain gut bacteria, including those related to hormone regulation and anxiety. These findings suggest that estradiol can enhance extinction and gut bacteria–related processes in ways that may support treatments of fear-related mental illness.

Fear extinction is a type of learning in which learned fear responses are reduced after repeated exposures to a stimulus that was previously paired with an aversive stimulus during fear conditioning. The processes driving these fear behaviors are impacted in individuals with anxiety and fear-based disorders, such as posttraumatic stress disorder (PTSD). Individuals with PTSD show worse fear extinction learning compared with trauma-exposed healthy control participants in human paradigms of fear-potentiated startle (1,2). Exposure therapy, the standard treatment for PTSD, relies on fear extinction processes. Gaining a better understanding of how fear extinction learning and memory can be enhanced may improve responses to exposure-based treatments for individuals affected by PTSD.

In contrast to human studies, rodent models of fear extinction provide a method to investigate the neurobiological mechanisms of fear expression and regulation more directly. In a typical auditory-cued fear conditioning and extinction paradigm, the animal learns to associate a previously neutral stimulus (tone) with an aversive unconditioned stimulus (US) (foot shock) during fear conditioning. After repeatedly pairing these stimuli, the animal learns to fear this cue, now the conditioned stimulus (CS), and displays fear, typically measured as freezing behavior. During fear extinction, the CS is repeatedly presented without the US. Freezing behavior gradually decreases as the animal learns that the threat is no longer associated with the CS. The previously acquired “fear” memory is inhibited by the new “safety” memory; this new safety learning is referred to as fear extinction. After fear extinction learning, a recall test is used to measure the strength of the new safety memory 24 hours later.

Fear extinction mechanisms have been studied extensively in male rodents (3), and while sex differences have been demonstrated in these mechanisms, it is still unclear why these differences exist. The role of sex hormones has been examined to help explain these differences, which has increased the inclusion of females in this area of research. Previous research has demonstrated a facilitative effect of estradiol (E2) on fear extinction consolidation; naturally cycling female rodents in proestrus, a period of high E2 levels, exhibit enhanced fear extinction recall 24 hours after training compared with female rats in metestrus, a low E2 phase (4, 5, 6). Exogenous administration of E2 or estrogen receptor agonists produces similar facilitative effects on fear extinction, while depletion of E2 through hormonal contraceptives or ovariectomy impairs fear extinction consolidation in female rats (7, 8, 9, 10). Human research has corroborated these findings; reduced E2 via administration of hormonal contraceptives impairs fear extinction memory in women (9). Therefore, the effect of E2 on fear extinction recall 24 hours after extinction acquisition has been well established, but the duration and mechanism of this effect remain uncertain. In the current study, we explored the persistence of E2’s facilitative effect 1 to 2 weeks following extinction training and the involvement of the gut microbiome at these time points.

The gut-brain axis has been increasingly connected to anxiety in both humans and animal research (11,12). However, there is conflicting evidence on how the gut microbiome influences fear extinction learning and memory. Chu *et al.* (13) reported that germ-free and antibiotic-treated male mice had impaired fear extinction learning compared with control mice. In contrast, Geary *et al.* (14) found that antibiotic administration did not significantly impair fear extinction learning or recall, although high E2 female mice exhibited resistance to the effects of antibiotic treatment (14). Moreover, there is evidence that enhancing the abundance of microbiota in the *Lactobacillaceae* and *Bifidobacteriaceae* families can have anxiolytic effects in male rodents (15,16).

The gut microbiome is also sensitive to changes in circulating ovarian hormone levels, with several prior studies demonstrating an increase in microbial diversity as a result of increased E2 (17, 18, 19, 20). Despite the bidirectional relationship observed between the gut microbiome and ovarian hormones, little research has focused on how this interaction contributes to the gut microbiome’s modulation of anxiety-like behavior. In the current study, we attempt to 1) determine whether the facilitative effects of E2 on fear extinction recall observed 24 hours later are also observed 1 to 2 weeks after extinction training and 2) identify changes in the gut microbiome after E2 administration and determine whether these changes are correlated with serum E2 levels and freezing behavior.

## Methods and Materials

### Animals

Fifty-seven adult female Sprague Dawley rats (8–10 weeks of age, 190–260 g, Charles River Laboratories) were housed in groups at the Animal Resources Core Facility in the Integrated Sciences Complex at the University of Massachusetts Boston. All animals were on an ad libitum diet of pellets (5P00 ProLab RMH 3000, LabDiet) and water with a 12-hour light/dark cycle. After 1 week of acclimation, all rats were handled daily for the duration of the study. The following procedures were reviewed and approved by the University of Massachusetts Boston Institutional Animal Care and Use Committee. All procedures abide by ARRIVE (Animal Research: Reporting of In Vivo Experiments) guidelines. A power analysis was performed using G∗Power software to determine the sample size needed to achieve 80% power and a medium effect size at a significance level of .05. Of the 57 rats, 14 rats were excluded from analyses for reasons detailed in the Supplement (Figure S1).

### Estrous Cycle Tracking

The estrous cycle of all rats was monitored via vaginal swabs from a minimum of 10 days prior to the start of the study until the end of the study (21). The rodent estrous cycle typically lasts 4 days, during which 4 phases can be identified: proestrus, estrus, metestrus, and diestrus. Rats were swabbed between 10 am and 12 pm.

### Blood Serum Collection and Analysis

Blood was collected at least 3 days prior to experimentation to establish baseline serum E2 concentrations at low and high estrogen phases of the estrous cycle and within 3 hours of each behavioral test. A 25-gauge needle was inserted into the lateral tail vein after warming the tail with water at approximately 40 °C for 2 to 3 minutes. Rats were briefly restrained in an absorbent pad or restraint tube during blood collection. Blood samples were left to clot for 90 minutes at room temperature (20 °C) before being centrifuged for 15 minutes at 1000*g*. Blood serum was extracted and stored at −80 °C until processed. Serum estradiol (E2) concentration was quantified using an enzyme-linked immunosorbent assay (Rat Estradiol ELISA, MyBioSource) according to the instructions provided.

### E2 Administration

17β-estradiol (15 μg/kg) was administered via a single subcutaneous injection (Sigma-Aldrich) in a sesame oil vehicle. Each rat was injected with 0.2-mL vehicle or E2 30 minutes before the start of extinction training. The E2 dose and timing of administration was used previously and shown to enhance learning and memory in female rats (5,7,8,22,23).

### Behavioral Testing

All behavior testing occurred in one of four 25 × 33 × 29 cm sound-proof chambers (Lafayette Instruments). Each chamber had a house light, video camera, speaker, indicator light, and steel bar floors, which were used to deliver the foot shock. Chambers were sanitized after each use with vital oxide disinfectant (Vital Solutions) or 70% ethanol.

Prior to behavioral testing, all animals had a 1-week acclimation period before 2 weeks of daily handling (Figure S1). Estrous cycle tracking and baseline fecal and serum sample collections occurred during this time. All rats underwent pre-exposure to their assigned chambers for 30 minutes a day for 3 days. The US during fear conditioning was a 0.5-second foot shock at 0.5 mA, coterminating with the indicator light and CS, a 30-second tone at 80 dB and 4 kHz. The concurrent activation of the CS tone and indicator light marked the beginning of a 30-second trial with intertrial intervals ranging from 80 to 280 seconds.

On day 1, animals underwent habituation (5 tone-alone trials) and fear conditioning (7 tone-shock paired trials) in estrus. Rats were assigned to treatment groups matched by the level of freezing during fear conditioning to control for pretreatment differences in acquisition. Extinction training (20 tone-alone trials) took place on day 2 while animals were in metestrus. Thirty minutes before extinction training, 21 rats were administered E2 (15 μg/kg), and 22 were administered vehicle (sesame oil). Experimenters were blinded to the treatment groups during data collection, and the blind was removed only for statistical analysis at the end of the study.

On day 3, during diestrus, the recent extinction recall test took place with 3 tone-alone trials. Thirteen vehicle-treated and 12 E2-treated rats underwent a remote extinction recall test during metestrus 1 to 2 weeks after extinction training. All animals underwent the remote extinction recall retest in the same estrous phase (metestrus) as they were in during fear extinction learning.

ANY-maze software was used to calculate freezing behavior during all trials (CS presentations) across all phases. Freezing behavior, a well-studied passive fear response in rodents, was defined as the absence of all voluntary movement, aside from respiration, for more than 0.2 seconds. Freezing was recorded in seconds for the entire duration of each trial and calculated as a percentage of the 30-second trial.

### 16S Ribosomal RNA Sequencing and Analysis of Gut Bacteria

To examine changes in the gut microbiota at specific points during the experimental timeline, fecal samples were collected at least 3 days prior to experimentation and within 3 hours of each behavioral testing phase. Fecal samples were obtained by placing rats in a plastic transport cage sterilized with 70% ethanol or caught in autoclaved 2-mL microcentrifuge tubes during handling between 10 am and 2 pm. Fecal samples were immediately placed into ice before being stored at −80 °C.

DNA was extracted from 75- to 220-mg fecal samples using the Qiagen QIAamp Fast DNA Stool Mini Kit (cat. #51604) according to the instructions included in the kit. All samples were sent to Wright Labs, LLC for bacterial analyses. An Invitrogen Qubit 4 Fluorometer and 1 × Qubit dsDNA High Sensitivity Assay Kit (Thermo Fisher Scientific) were used for sample quantification. The polymerase chain reaction amplification methods used for 16S ribosomal RNA (rRNA) sequencing and species-level identifications have been described previously (24, 25, 26, 27). Please see the Supplement for details.

### Statistical Analysis

Behavioral data from habituation, conditioning, and extinction were averaged in 2-trial blocks for analysis and graphing. Freezing behavior during the first CS presentation (trial 1 of habituation) was not included in the overall habituation average due to the novelty of the CS during this trial. Similarly, freezing behavior during the sixth trial of habituation and conditioning, the first US presentation, was not included in the average of conditioning due to the novelty of the US during this trial. Freezing behavior during recent and remote fear extinction recall were averaged as 2 separate data points, respectively. Rats whose average freezing behavior during trials 7 to 12 of habituation and conditioning fell below 25.0% failed to condition and were excluded from further analysis. Behavioral videos that were not suitable for scoring by Any-Maze behavior tracking software were manually scored by 3 trained raters, intraclass correlation coefficient (ICC) >0.95. Statistical analysis software SPSS (IBM Corp.) was used to conduct analyses on the behavioral data. Mixed-design repeated-measures analysis of variance (ANOVA) was used to examine average freezing behavior between the control and E2-treated group during habituation, conditioning, and extinction. Because recent and remote fear extinction recall were both averaged as one data point, one-tailed independent-samples *t* tests were used to examine group differences in freezing behavior during both behavioral phases. Corrected values were reported when equal variances were not assumed.

A linear mixed-effects model conducted with SPSS was used to examine the differences in serum E2 concentration between groups and across 6 time points: baseline (proestrus), baseline (metestrus), habituation and conditioning (day 1), extinction training (day 2; 30 minutes after E2 or vehicle injection), recent recall (day 3), and remote recall. 16S rRNA sequencing analyses are detailed in the Supplement.

## Results

### Serum E2 Concentration

Serum was collected from naturally cycling female rats during metestrus and proestrus at least 3 days prior to experimentation and within 3 hours of each behavioral test. On the extinction training day, serum was collected 30 minutes after vehicle or E2 administration, and extinction training began immediately after. Serum E2 concentration was significantly higher in E2-treated compared with vehicle-treated rats only during extinction training (*F*_5,26.767_ = 7.320, *p* < .001) (Table S1).

### Behavior

**Figure 1:**
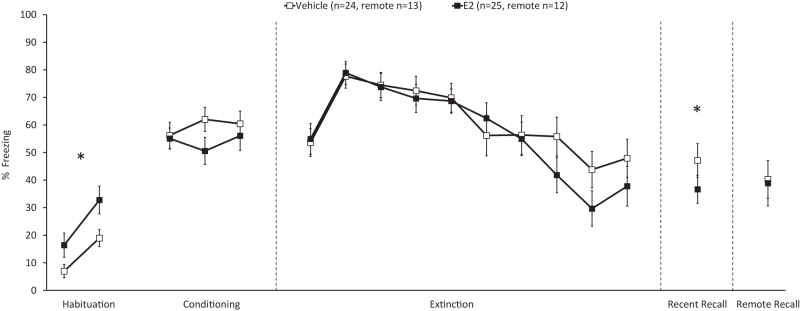
Percent freezing averages during habituation and conditioning on day 1, extinction training on day 2, recent recall on day 3, and remote recall 1 to 2 weeks after the extinction session. A one-sided independent-samples *t* test revealed that the administration of 15 μg/kg of E2 30 minutes prior to extinction training significantly decreased freezing behavior 24 hours later at the recent fear extinction recall test (*t*_41_ = 1.680, *p* = .05), but not remote (*p* > .05). ∗*p* ≤ .05. White squares indicate control, and black squares indicate E2. E2, estradiol.

All rodents (*N* = 43) underwent fear conditioning in estrus on day 1, fear extinction training in metestrus on day 2, and recent fear extinction recall in diestrus on day 3. A subset of rodents (*n* = 25) underwent remote fear extinction recall 9 to 16 days after extinction training (Figure S1). Freezing behavior across all behavioral tests was averaged into 2-trial blocks, omitting the first trial of habituation and the first trial of conditioning. Freezing behavior during the 3 trials of recall was averaged as one data point. A mixed-design repeated-measures ANOVA revealed that there was a main effect of block during habituation (*F*_1,41_ = 17.929, *p* < .001), a main effect of group (*F*_1,41_ = 7.001, *p* = .012), and no group × block interaction (*F*_1,41_ = 0.956, *p* = .334). These results indicated a significant increase in freezing across the habituation phase and greater freezing levels in the E2-treated rats compared with vehicle–treated rats during habituation (Figure 1). A main effect of block (*F*_2.462,100.955_ = 6.914, *p* < .001) was observed during the conditioning phase, with a block × group interaction (*F*_2.462,100.955_ = 5.091, *p* = .002), and no main effect of group (*F*_1,41_ = 0.659, *p* = .421), which indicated that freezing significantly increased during conditioning in both groups (Figure 1). For the extinction training phase, there was an effect of block (*F*_9,351_ = 13.671, *p* < .001) because freezing decreased across extinction trials but no effect of group (*F*_1,39_ = 0.130, *p* = 7.20) or block × group interaction (*F*_4.959,193.397_ = 0.712, *p* = .614) (Figure 1). Although the E2-treated group had significantly greater freezing behavior during habituation prior to E2 treatment, there were no group differences observed in freezing behavior during conditioning and extinction. A one-tailed independent-samples *t* test revealed that the E2-treated group had significantly lower freezing during recent recall than the vehicle-treated group (*t*_41_ = 1.680, *p* = .05), but not during remote recall (*t*_23_ = 0.658, *p* > .05) (Figure 1).

Additional analyses were performed to identify other possible E2 effects but showed that E2 administration did not significantly impact estrous cycle length, body weight, and locomotive behavior (e.g., darting behavior, distance traveled, and speed) (see the Supplement, Figure S2).

### Gut Microbiome Composition

Fecal boli were collected at least 3 days prior to experimentation and within 3 hours of each behavioral test. 16S rRNA sequencing was used to examine differences in abundance and diversity of several species of gut microbiota. After filtering out samples with amplicon sequencing variants below 6400, gut microbiota analysis was completed with 137 samples.

**Figure 2:**
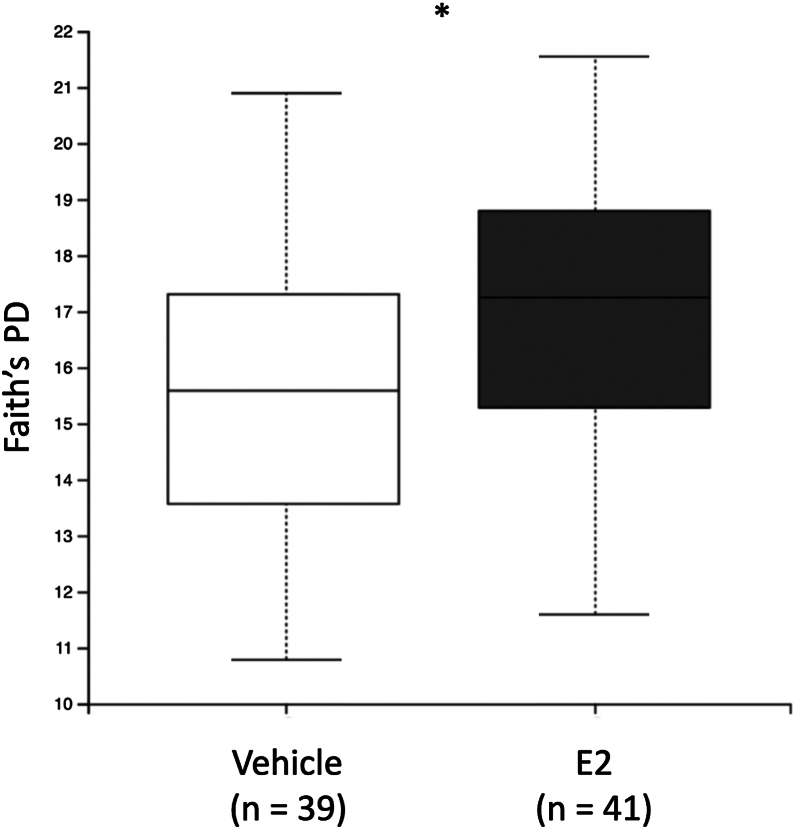
Faith’s PD was used as a measure of alpha diversity (28). Due to the non-normal distribution of data, a Kruskal-Wallis test was performed to analyze the effect of treatment (vehicle vs. E2) on alpha diversity (Faith’s PD) of samples collected after vehicle or E2 administration (i.e., extinction, recent recall, and remote recall time points). E2-treated rats had significantly greater Faith’s PD compared with vehicle-treated rats (*H*_1_ = 5.95, *p* = .015). ∗*p* < .05. E2, estradiol; PD, phylogenetic diversity.

Faith’s phylogenetic diversity (PD) was used as a measure of alpha diversity (28). A Kruskal-Wallis test revealed a significant increase in Faith’s PD of samples from E2-treated rats collected at the extinction, recent recall, and remote recall time points (*H*_1_ = 5.95, *p* = .015) (Figure 2). Due to the nonparametric distribution of these data and limitations imposed by missing data, a rank-based linear mixed model was conducted to examine within-subject effects of treatment (vehicle vs. E2) and time point (baseline, conditioning, extinction, recent recall, and remote recall) on Faith’s PD (Figure S3). The analysis revealed no significant main effect of treatment (*F*_1,40.369_ = 2.0243, *p* = .169), indicating that overall alpha diversity did not differ significantly between the vehicle- and E2-treated groups (Table S2). There was also no significant main effect of time point, with no time point differing significantly from baseline (*p* > .05). Additionally, the treatment × time point interaction was not significant (*p* > .05), suggesting that changes in alpha diversity across time did not differ between treatment groups. The model accounted for a substantial proportion of variance when subject-level random effects were included (conditional *R*^2^ = 0.602), although fixed effects alone explained relatively little variance (marginal *R*^2^ = 0.070). The ICC of 0.57 indicated substantial within-subject consistency across time points.

Beta diversity, the overall similarity between microbial communities represented in fecal samples collected from vehicle- and E2-treated rats, was measured using weighted UniFrac. For both nonparametric and multivariate data, QIIME2’s longitudinal pairwise-distances test was used to examine the magnitude of the weighted UniFrac distance between each respective time point relative to baseline for both groups. There were no significant differences in the magnitude of within-individual beta diversity shifts observed at any time point between vehicle- and E2-treated rats (*p* > .05).

**Figure 3:**
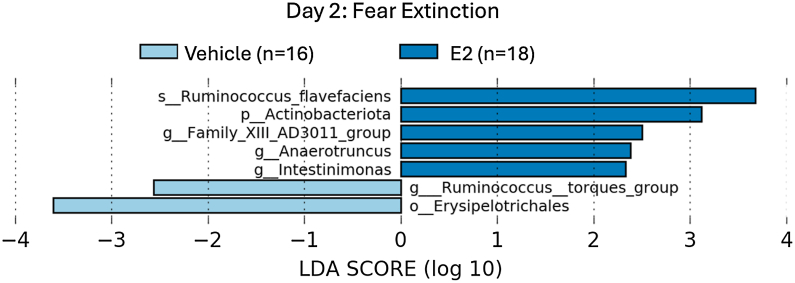
Biomarker analysis was performed using LDA effect size to identify taxa that had significantly different abundances during extinction acquisition on day 2 (LDA ≥ 2.0). E2, estradiol; LDA, linear discriminant analysis.

**Figure 4:**
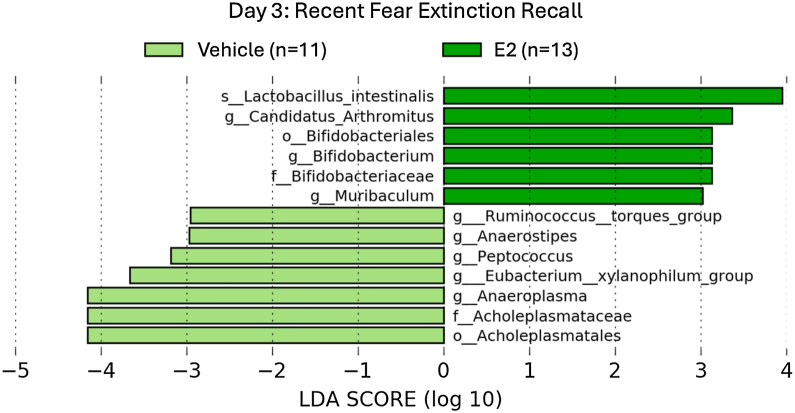
Biomarker analysis was performed using LDA effect size to identify taxa that had significantly different abundances during recent extinction recall on day 3 (LDA ≥ 2.0). E2, estradiol; LDA, linear discriminant analysis.

**Figure 5:**
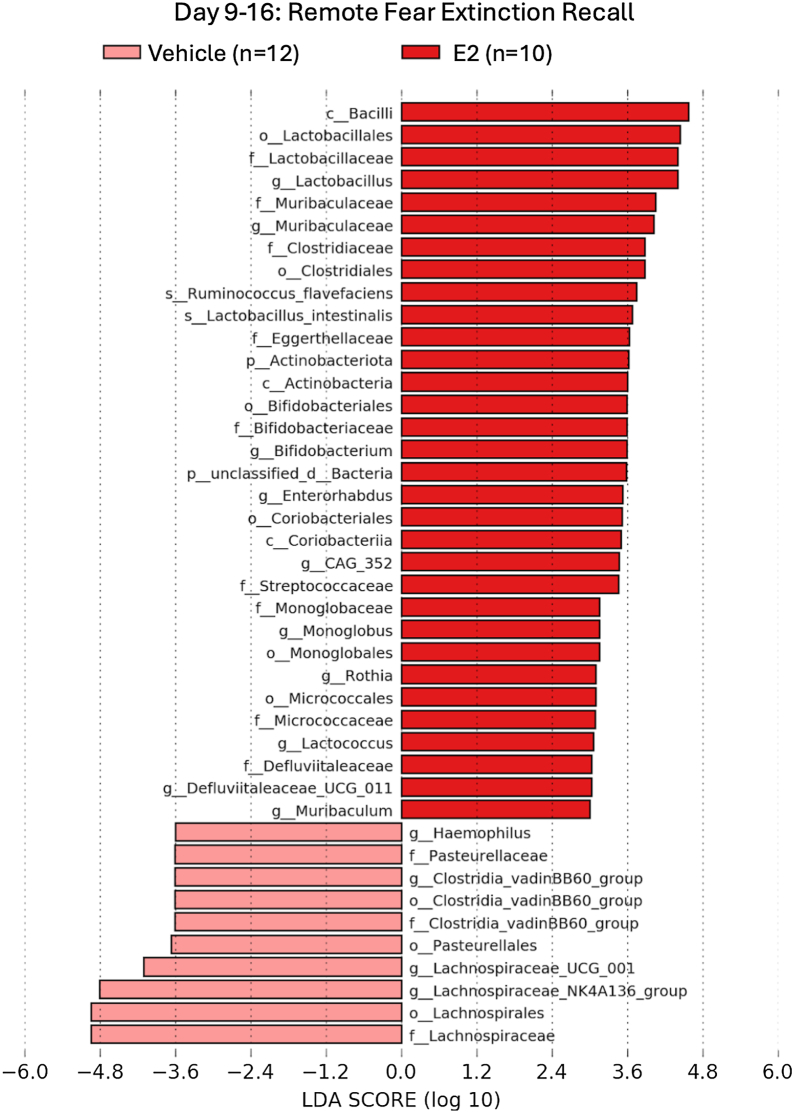
Biomarker analysis was performed using LDA effect size to identify taxa that had significantly different abundances during remote extinction recall 1 to 2 weeks after vehicle or E2 administration and extinction training (LDA ≥ 2.0). Several bacterial families within the Firmicutes phylum (Lactobacillaceae, Clostridiaceae, Streptococcaceae) were enriched in E2-treated rats at remote fear extinction recall. *Lactobacillus intestinalis* was enriched in the E2-treated group at both recall time points. E2, estradiol; LDA, linear discriminant analysis.

Linear discriminant analysis effect size (LEfSe) was used to identify taxa down to the species level with significantly different abundance. Gut taxa with significantly different abundance between treatments in one time point (Kruskal-Wallis, *p* ≤ .05) and with a log (linear discriminant analysis [LDA]) score of ≥2.0 were determined to be differentially enriched (Figure 3, Figure 4, Figure 5). Nine unique bacterial families were significantly enriched in the E2-treated rats at remote recall: Lactobacillaceae, Muribaculaceae, Clostridiaceae, Eggerthellaceae, Bifidobacteriaceae, Streptococcaceae, Micrococcaceae, Monoglobaceae, and Defluviitaleaceae (Figure 5). Four of these families are members of the Firmicutes phylum (Lactobacillaceae, Clostridiaceae, Monoglobaceae, and Defluviitaleaceae), and one is a member of the Bacteroides phylum (Muribaculaceae). Firmicutes and Bacteroides are the most prominent estrogen-regulating phyla (29).

In addition, we examined the predictive relationships between gut microbiome composition, fear behavior, and serum E2 concentration (pg/mL) using Spearman’s rho (*r*_s_); the threshold of all correlations was set at *r*_s_ ≥ 0.4 and *p* < .05 (Table 1). The abundance of the *Ruminococcus* genus on the day of extinction was positively correlated with serum E2 levels in the E2-treated group 30 minutes after E2 administration but not in the vehicle-treated group (*r*_s_ = 0.77, *p* = .005) (Table 1). In addition, the abundance of the *Alistipes* genus on the day of recent recall was negatively correlated with freezing behavior during recent recall in E2-treated rats but not in vehicle-treated rats (*r*_s_ = −0.58, *p* = .022) (Table 1).

**Table 1:** Significant Relationships Between Gut Taxa, Behavior, and Serum E2 Concentration Within Each Treatment Group (Control, E2)

Treatment	Freezing Behavior	Extinction Retention Index	Serum E2 Concentration, pg/mL
Vehicle (*n* = 16) (Extinction)	–	N/A	s_Monoglobus uncultured species (*r*_s_ = 0.71)∗∗ s_Ruminococcus uncultured species (*r*_s_ = 0.71)∗∗ s_Rhodospirillales uncultured species (*r*_s_ = 0.70)∗∗ g_Lachnospiraceae_UCG-001 (*r*_s_ = −0.68)∗ s_Lachnospiraceae uncultured species (*r*_s_ = −0.66)∗ s_Tyzzerella uncultured species (*r*_s_ = −0.65)∗ s_Muribaculaceae uncultured species (*r*_s_ = 0.61)∗ g_Roseburia (*r*_s_ = −0.60)∗ s_Oscillospiraceae UCG-005 uncultured species (*r*_s_ = −0.60)∗
Vehicle (*n* = 11) (Recent Recall)	g_Lachnospiraceae uncultured genus (*r*_s_ = −0.66)∗ o_RF39 uncultured (*r*_s_ = 0.63)∗	g_Monoglobus (*r*_s_ = −0.66)∗∗ s_Clostridia_UCG-014 uncultured species (*r*_s_ = −0.61)∗ s_Lachnospiraceae_UCG-001 uncultured species (*r*_s_ = 0.61)∗ s_RF39 uncultured species (*r*_s_ = −0.64)∗	s_Rhodospirillales uncultured species (*r*_s_ = −0.85)∗∗ s_Oscillibacter unidentified species (*r*_s_ = 0.83)∗ s_Tuzzerella uncultured species (*r*_s_ = 0.81)∗ g_Bilophila (*r*_s_ = −0.76)∗ g_Ruminococcus (*r*_s_ = 0.74)∗ s_Eubacterium coprostanoligenes group unidentified species (*r*_s_ = 0.74)∗ s_Oscillospiraceae NK4A214_group uncultured species (*r*_s_ = −0.74)∗ s_Monoglobus uncultured species (*r*_s_ = 0.73)∗ s_Desulfovibrionaceae uncultured species (*r*_s_ = −0.73)∗ g_Oscillospiraceae NK4A214_group uncultured genus (*r*_s_ = 0.73)∗ s_Lachnospiraceae_UCG-001 uncultured species (*r*_s_ = 0.73)∗
Vehicle (*n* = 12) (Remote Recall)		s_Oscillospiraceae uncultured species (*r*_s_ = 0.58)∗ s_Muribaculaceae uncultured species (*r*_s_ = 0.58)∗ s_Roseburia uncultured species (*r*_s_ = 0.59)∗ s_Candidatus Saccharimonas uncultured species (*r*_s_ = −0.71)∗∗ s_Acetatifactor uncultured species (*r*_s_ = 0.68)∗ s_Ruminococcus unidentified species (*r*_s_ = −0.62)∗ g_Oscillibacter species (*r*_s_ = −0.66)∗	g_Lachnoclostridium (*r*_s_ = −0.93)∗∗ g_Streptococcus (*r*_s_ = −0.91)∗∗ s_Oscillospiraceae uncultured species (*r*_s_ = 0.89)∗∗ s_Clostridia vadinBB60 group unidentified species (*r*_s_ = −0.89)∗∗ s_Muribaculaceae uncultured species (*r*_s_ = −0.87)∗ s_Rhodospirillales uncultured species (*r*_s_ = −0.85)∗ g_Clostridia vadinBB60 group unidentified genus (*r*_s_ = 0.82)∗ s_Alistipes unidentified species (*r*_s_ = −0.81)∗ s_Ruminococcaceae CAG-352 uncultured species (*r*_s_ = −0.80)∗ s_Lachnospiraceae UCG-006 uncultured species (*r*_s_ = −0.79)∗ s_Ruminococcus unidentified species (*r*_s_ = −0.79)∗ o_Oscillospirales (*r*_s_ = 0.77)∗
E2 (*n* = 18) (Extinction)		N/A	g_Ruminococcus (*r*_s_ = 0.77)∗∗ s_Ruminiclostridium uncultured species (*r*_s_ = −0.77)∗∗ s_Prevotellaceae_UCG-001 uncultured species (*r*_s_ = 0.76)∗∗ s_Ruminococcaceae uncultured species (*r*_s_ = −0.76)∗∗ g_Eubacterium coprostanoligenes group unidentified genus (*r*_s_ = 0.74)∗∗ s_Enterorhabdus uncultured species (*r*_s_ = −0.69)∗ s_Rhodospirillales uncultured species (*r*_s_ = 0.68)∗ s_Ruminococcus unidentified species (*r*_s_ = −0.62)∗ s_Lachnospiraceae FCS020 group unidentified mouse gut species (*r*_s_ = −0.61)∗ s_Lachnospiraceae A2 uncultured species (*r*_s_ = −0.61)∗ s_Eubacterium coprostanoligenes group unidentified species (*r*_s_ = −0.60)∗
E2 (*n* = 13) (Recent Recall)	g_Alistipes (*r*_s_ = −0.58)∗	s_Lachnospiraceae_UCG-006 uncultured species (*r*_s_ = −0.53)∗ s_Defluviitaleaceae_UCG-011 uncultured species (*r*_s_ = 0.69)∗∗ s_Peptococcaceae uncultured species (*r*_s_ = −0.57)∗ f_Erysipelatoclostridiaceae (*r*_s_ = −0.67)∗∗ s_Lachnospiraceae NK4A136 group Trichinella pseudospiralis (*r*_s_ = −0.52)∗ g_Defluviitaleaceae_UCG-011 (*r*_s_ = −0.58)∗ f_Prevotellaceae (*r*_s_ = 0.51)∗	s_Clostridia UCG-014 uncultured species (*r*_s_ = −0.95)∗∗∗ s_Prevotellaceae UCG-001 uncultured species (*r*_s_ = −0.85)∗ s_Monoglobus unidentified species (*r*_s_ = −0.80)∗ g_Butyricicoccus (*r*_s_ = −0.80)∗ s_Izemoplasmatales unidentified species (*r*_s_ = −0.78)∗
E2 (*n* = 10) (Remote Recall)		g_Ruminococcus (*r*_s_ = −0.72)∗ o_Oscillospirales (*r*_s_ = 0.75)∗ s_Monoglobus uncultured species (*r*_s_ = −0.83)∗∗ g_Clostridia vadinBB60 group uncultured genus (*r*_s_ = 0.75)∗ s_Erysipelotrichaceae uncultured species (*r*_s_ = −0.77)∗∗ s_Anaerovorax uncultured species (*r*_s_ = 0.76)∗∗ s_Ruminococcus unidentified species (*r*_s_ = −0.68)∗ s_Akkermansia uncultured species (*r*_s_ = −0.74)∗ s_Oscillibacter unidentified species (*r*_s_ = 0.69)∗	

Genus, order, species, and Spearman’s rho are indicated by g, o, s, and *r*_s_, respectively.

∗*p* ≤ .05, ∗∗*p* ≤ .01, ∗∗∗*p* ≤ .001.

## Discussion

In the current study, we examined the effects of exogenous E2 on fear extinction memory and gut microbiota. The main findings of this study were that 1) E2 administration before extinction training enhanced recent but not remote extinction recall, and 2) E2 administration enriched estrogen-regulating and anxiety-related gut taxa.

The current findings add to a large body of evidence that increased E2 levels in female rats during extinction acquisition lead to enhanced fear extinction recall 24 hours later (4, 5, 6, 7, 8). However, this facilitation does not extend to the remote recall time point 1 to 2 weeks after fear extinction learning. It is possible that the acute facilitative effects of E2 on fear extinction consolidation are the result of the rapid effects of estrogen receptor (ER) signaling. 17β-estradiol acts on several receptors; estrogen receptor alpha, estrogen receptor (ERβ), and G protein–coupled estrogen receptor (GPER1/GPR30), which can initiate rapid and transient intracellular signaling pathways. These receptors are expressed in fear extinction–related regions of the brain, namely the medial prefrontal cortex (mPFC), hippocampus, and amygdala (30, 31, 32, 33, 34, 35). E2’s enhancing effects on other forms of memory in female, but not male, rodents has been shown to depend on the activation of nongenomic, ER-mediated cell signaling pathways (36,37). However, the mechanisms underlying the effects of E2 on fear extinction memory in females are not fully understood. Prior research has shown that exogenous E2 administration alters the activity of the infralimbic (IL) cortex of the mPFC and central amygdala (7,8). Furthermore, Cover *et al.* (38) suggested that ER activation of the MAPK/ERK (mitogen-activated protein kinase/extracellular signal-regulated kinase) signaling pathway in the IL cortex is a possible mechanism through which E2 enhances fear extinction recall in females.

The gut microbiome plays a role in modulating fear extinction circuitry and behavior, as it has been suggested that gut dysbiosis impairs fear extinction in male mice (13). Although microbial diversity did not differ significantly between treatment groups (Figure S3), specific gut microbiota were found to be correlated with freezing behavior and serum E2 levels. The *Alistipes* genus was negatively correlated with freezing behavior during recent fear extinction recall in E2-treated rats (*r*_s_ = −0.58, *p* = .022) (Table 1). Previous studies have also described the influence of ovarian hormone status on *Alistipes*, where *Alistipes* was enriched in ovariectomized female mice (39). *Alistipes* was also enriched in Korean adults who exhibited low levels of anxiety and depression, indicating an association between *Alistipes* and psychiatric disorders (40). The abundance of *Lactobacillus intestinalis*, a gut microbe also linked to reduced anxiety and enriched after progesterone supplementation in ovariectomized female rats, was significantly increased in E2-treated rats at recent and remote extinction recall (20). Together, E2 treatment seems to specifically affect gut bacteria that may be involved in anxiety and fear-related behavior.

Several studies have shown that increased E2 in humans and rodents is associated with greater gut bacterial diversity, which seems to be related to better health; however, the mechanisms through which E2 influences the gut are not well understood (17, 18, 19). One hypothesis is associated with ERβ-mediated effects on gut permeability (41,42). The lining of the gut is a permeable barrier between gut bacteria and the internal milieu. Disruption of this barrier is associated with increased inflammation, gastrointestinal (GI) disorders, and imbalanced gut microbiota (43). ERβ activation via E2 administration in male mice reduces GI symptoms, damage to the intestinal barrier, and inflammation (44). In support of this, male ERβ knockout mice had reduced gut microbiome diversity, more anxiety-like behavior, and greater severity of GI symptoms (45). However, these studies were conducted only in male mice.

Another possible mechanism underlying the effects of E2 on gut microbial diversity is E2’s influence on gut motility. Gut motility, the movement of food through the GI system, is regulated by regions of the enteric and central nervous system that are known to have ERs, such as the hypothalamus and vagus nerve (46, 47, 48, 49, 50, 51, 52). E2 supplementation inhibits gut motility, and decreased gut mobility is linked to increased gut microbial richness and diversity (53,54). E2 can reduce food intake, which in turn may influence gut microbiota (55). Although food consumption was not measured directly, our data indicate no significant difference in body weight between vehicle-treated and E2-treated rats during extinction, when E2 levels are the highest or at any other time point (Figure S2). Taken together, the relationship between E2 and gut microbial diversity could be mediated through the effects of E2 on gut motility and/or gut permeability.

A limitation of this work is that the remote recall time point took place 1 to 2 weeks after extinction training. There may be undetected differences within this time range that might have been observed if the remote group was within a smaller range of time from injection; however, we did not have sufficient sample sizes when dividing this group into 1- and 2-week postextinction time points to make comparisons. Follow-up studies may improve time point distinctions. Finally, this study also revealed that the E2 dose administered (15 μg/kg) induced supraphysiological levels of circulating E2. The average serum E2 concentration (pg/mL) 30 minutes after administration was significantly greater than the average E2 concentration at proestrus (Table S1). However, this 15 μg/kg dose and 30-minute timing of administration has been used and found to be effective in improving memory in several other studies (5,7,8,20,21). Additionally, our data indicated no significant effect of E2 administration on normal estrous cycling and average estrous cycle length (see the Supplement).

### Conclusions

Understanding the influence of estrogen on the long-term maintenance of fear extinction memory is crucial given the increased prevalence of fear-related disorders in women. Women are also at a greater risk of developing GI disorders, but the interactions of estrogen and the gut microbiome are still not fully understood. Our research demonstrates that fear extinction recall and the gut microbiome are both impacted by E2. E2 administration prior to extinction learning enhanced fear extinction recall 24 hours later; however, facilitative effects of E2 did not extend to 1 to 2 weeks after extinction learning. More research is needed to understand why this effect is not durable over time. Finally, E2 significantly increased the abundance of estrogen-reactive and anxiety-related taxa. Taken together, the current findings expand our understanding of how E2 influences aversive learning and memory and impacts related gut microbiota.

## Supplementary Material

Supplemental Methods, Results, Figures S1–S3, Tables S1–S2

Key Resources Table
