# Supplementary material for: Acute 17β-Estradiol Administration Enhances Fear Extinction Memory and Alters Gut Microbiota in Female Rats
Source: Biol Psychiatry Glob Open Sci. 2025 Sep 26;6(1):100620. doi: 10.1016/j.bpsgos.2025.100620 (PMC12664449; doi:10.1016/j.bpsgos.2025.100620)
Supplement: Supplemental Methods, Results, Figures S1–S3, Tables S1–S2 [file mmc1.pdf]

SUPPLEMENTARY INFORMATION

Acute 17β-Estradiol Administration Enhances Fear Extinction Memory and Alters Gut Microbiota in Female Rats

Hartsgrove *et al.*

Supplementary Materials

Gut Microbiome Sequencing

The 16S rRNA sequencing of gut microbiota was conducted by Wright Labs, LLC (Huntingdon, Pennsylvania) using PCR amplification methods described in Chen See et al. (2021) and Sapp et al. (2022) (1, 2). As detailed in the Earth Microbiome Project protocol (3), all 16S rRNA illumina-tag PCR reactions were performed on the extracted DNA. After the PCR products were pooled, they were purified using a 2% agarose QIAquick Gel Purification Kit (Qiagen, Frederick, MD, USA). Using the Agilent 2100 BioAnalyzer and Agilent DNA High Sensitivity DNA kit (Agilent Technologies, Santa Clara, CA, USA), quality of the gel purified pool was checked before sequencing, which was performed by Wright Labs, LLC (Huntingdon, PA, USA). QIIME2 was used for data processing and analyses (4). Data quality was assessed in QIIME2 using Phred q scores, and VSEARCH calculated the cumulative expected errors at each position (5). Based on these quality metrics, forward and reverse reads were truncated to a length of 200, both with a maximum expected error of 0.5, using QIIME2's DADA2 pipeline (6). The DADA2 pipeline in QIIME2 was also used to merge forward and reverse reads and then to classify the remaining sequences as amplicon sequence variants (ASVs).

Taxonomic information of ASVs was determined using representative sequences, as in Sapp et al. (2022) (2). The SILVA Database Version 138 (0.24.1) was used to match sequences to taxonomic information. ASVs determined to be chloroplasts or mitochondria were removed as possible eukaryotic contaminants rather than bacterial signal. Samples containing fewer than 1,000 sequences after filtration were excluded from the ASV table.

Metrics of alpha diversity, Faith's Phylogenetic Diversity (7), Pielou's Evenness (8), and Observed ASVs (9), were calculated by subsampling the ASV table at 10 different depths with a range from 640 up to 6400 sequences. At each of the 10 different depths of subsampling, 20 iterations were performed to calculate alpha diversity. A rarefaction plot was then used to confirm the diversity calculated approached an asymptote and slope decreased in response to increasing depth. Averages calculated for the greatest depths were used to analyze differences in alpha diversity across behavioral timepoints and between vehicle and estradiol treatment groups [Kruskal-Wallis test; $p \leq 0.05$].

The ASV table was normalized using cumulative sum scaling to reduce differences between samples due to sequencing depth (10). Beta diversity analyses were then conducted using the normalized table. Distances between samples were determined using the Weighted Unifrac metric based on the normalized ASV table and rooted phylogenetic tree (11). A Principal Coordinates Analysis plot (PCoA) was used to represent the resulting distance matrix. A PERMANOVA was used to analyze differences based on treatment group and time point ($p \leq 0.05$).

Finally, the ASV table was collapsed to level 7 (species) and normalized with the counts per million (CPM) method. Bacterial taxa that had significantly different abundances between groups were identified using a linear discriminant analysis effect size (LEfSe) analysis (12). Only taxa identified as having significantly different abundance (log(LDA) score > 2.0) were reported [Kruskal-Wallis, $p \leq 0.05$].

Behavior Analysis

Fear retention index (FRI), the amount of fear learning from day 1 observed during extinction learning on day 2, was calculated by subtracting the average freezing during the last block of conditioning from the first block of extinction. There were no significant differences in fear retention index, as revealed by independent samples t-test [$t(41) = 0.299$, $p = 0.393$]. Extinction retention index (ERI), the amount of extinction learning from day 2 observed during recent and remote extinction recall, was calculated by subtracting the average freezing during recall from the average freezing during the last two blocks of extinction. No differences were observed in extinction retention index (ERI) during recent fear extinction recall [$t(39)$, = -0.509, p = 0.307] or remote fear extinction recall [$t(21)$, = -0.370, p = 0.358].

E2 Effects on Estrous Cycle

The estrous cycle of all animals was tracked daily by trained researchers and found to not be significantly impacted by the exogenous E2/vehicle administration that took place on the day of extinction training. The average number of days between extinction training and the remote recall time point for vehicle-treated rats was 10.16 days (SEM=0.65) and the average number of full cycles during this time was 2.23 (SEM=0.65). Similarly, the average number of days between extinction training and the remote recall time point for E2-treated rats was 9.92 days (SEM=0.88) and the average number of full cycles during this time was 2.25 cycles (SEM=0.22). Further, the average cycle length – as measured by the number of days in between estrus – did not differ between groups. The average cycle length of vehicle-treated female rats was 4.37 days (SEM=0.17) and the average cycle length for E2-treated rats was 4.08 days (SEM=0.20). The estrous cycle of adult female Sprague Dawley rats is known to typically span 4 days, with little

variation. The E2-treated females exhibited normal estrous cycling through the proestrus, estrus, metestrus, and diestrus phases.

E2 Effects on Locomotion

E2 has also been found to increase locomotor activity (13, 14), which may confound the use of freezing behavior as a measurement of recovered fear during extinction training and recall. To investigate this, we analyzed the average distance travelled (m) and average speed (m/s) of each rat during trials (CS presentation) and inter-trial intervals during the extinction session. Using one-sided independent samples t-tests, we found no significant differences in the average distance travelled during extinction trials [t(39) = 0.349, p = 0.365] or extinction ITIs [t(39) = 0.299, p = 0.383]. In addition, we did not find significant differences in the average speed during extinction trials [t(39) = 0.349, p = 0.365] or extinction ITIs [t(39) = 0.522, p = 0.302]. We did find that E2-treated rats had a significantly greater average distance travelled during recent recall ITIs [t(37) = -1.848, p = 0.036] and a significantly greater average speed during recent recall ITIs [t(41) = -1.967, p = 0.028, but no significant differences in the distance travelled during recent recall trials [t(37) = -0.943, p = 0.176] or in average speed during recent recall trials [t(41) = -0.925, p = 0.18]. This result suggests that there may be an effect of E2 on locomotion 24 hours after administration, but it is not present during CS presentations of recent recall or during extinction training. Finally, we found no significant differences in the average distance travelled during remote recall trials [t(23) = -0.477, p = 0.319], distance travelled during remote recall ITIs [t(23) = -0.351, p = 0.364], speed during remote recall trials [t(23) = -0.455, p = 0.327], or speed during remote recall ITIs [t(23) = 0.186, p = 0.427].

In addition to freezing behavior, darting behavior was examined using the criteria set forth by Gruene et al. (15); a rat would be considered a “darter” if there was at least one occurrence of darting (velocity >23.5cm/s) during fear conditioning trials 8-12. We identified 1 vehicle-treated darter and 3 E2-treated darters. In addition to this, and in line with the findings from Gruene et al. (2015), darting behavior rarely took place beyond fear conditioning. The average darting count for both groups is <1 during habituation, extinction, recent recall, and remote recall, respectively. Since darting behavior occurs infrequently and primarily during fear learning, our study lacked the sample sizes needed to adequately analyze this behavior. For these reasons, we did not focus on darting behavior as a representation of fear response in this paper.

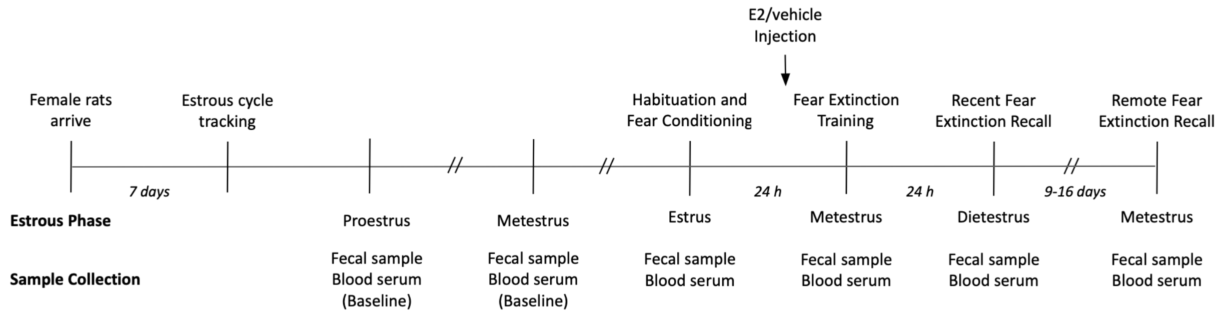

Figure S1. Timeline of experimental paradigm. Before any behavioral tests, rats were handled and swabbed daily for two weeks. During this time, baseline fecal and serum samples were collected, and rats were pre-exposed to their assigned chambers for 30 minutes/day for three consecutive days. All rats were in estrus, a low estrogen phase on the first day of the three-day behavioral paradigm. During the first day, habituation and fear conditioning took place; a tone (CS) was presented alone for five trials and then paired with a co-terminating 0.5s foot shock at 0.5mA (US) for seven trials. On day two, 24h later, rats underwent extinction training in the metestrus phase. Thirty minutes prior to extinction training rats received a subcutaneous injection of E2 (15µg/kg) or vehicle (sesame oil). During extinction training the un-paired CS was presented 20 times. Recent recall took place on day three, 24h after extinction training while all animals were in diestrus. Remote recall took place 1-2 weeks after extinction training. Both extinction recall tests included three unpaired CS presentations. Blood and fecal samples were collected immediately before all behavioral tests. Perfusions took place 1h after extinction recall tests. Fourteen of the fifty-seven rats were excluded from data analyses for the following reasons: failure to condition (n=7), injury (n=3), and failure to complete remote extinction recall within 1-2 weeks after extinction training (n=4). Forty-three rats were included in behavioral analyses, and 137 fecal samples were included in gut microbiota analyses.

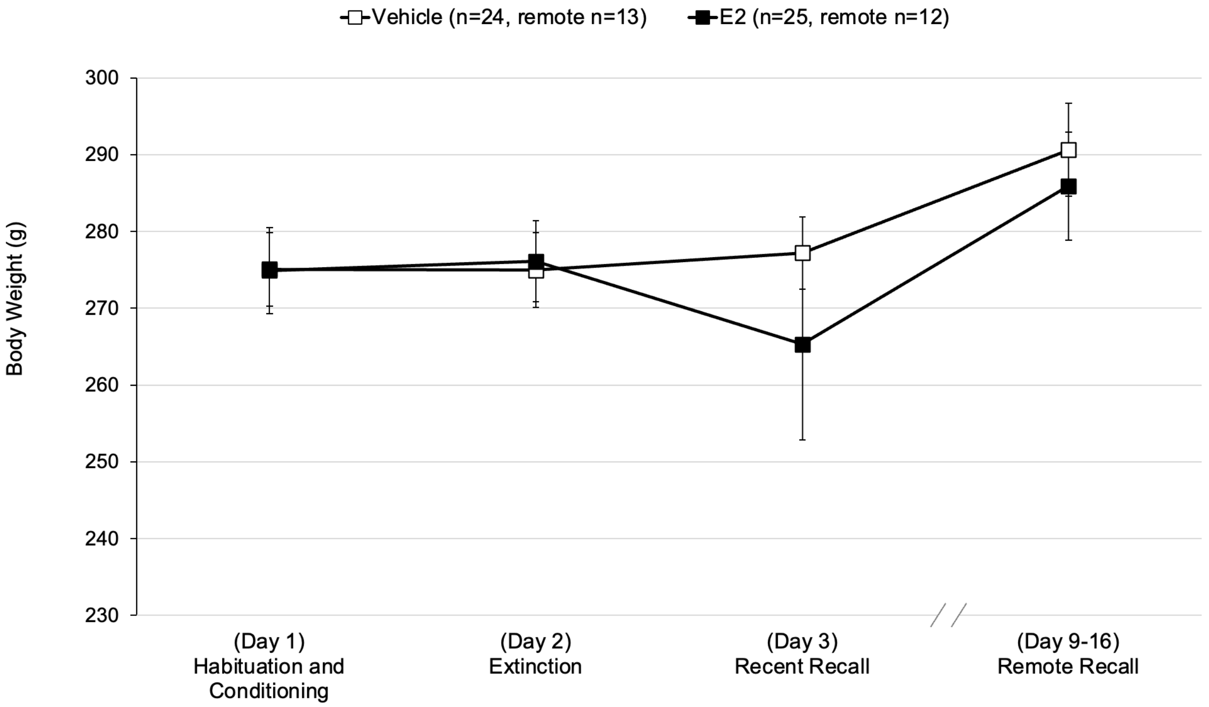

Figure S2. Line graph of body weights (g) of all female rats across the experimental paradigm. The body weights of all animals were measured daily starting one week after their arrival until the day of sacrifice. A 2x2 repeated measures ANOVA was used to examine the effects of time and treatment (vehicle vs. E2) on body weight. There was no significant effect of treatment on body weight or treatment x time interaction, but there was a significant effect of time [$F\,(3, 69) = 17.815$, $p < 0.001$]. Post-hoc pairwise comparisons revealed that the body weights of vehicle-treated rats at remote recall were significantly greater than those at habituation/conditioning, extinction, and recent recall, respectively, but this was not seen in E2-treated rats.

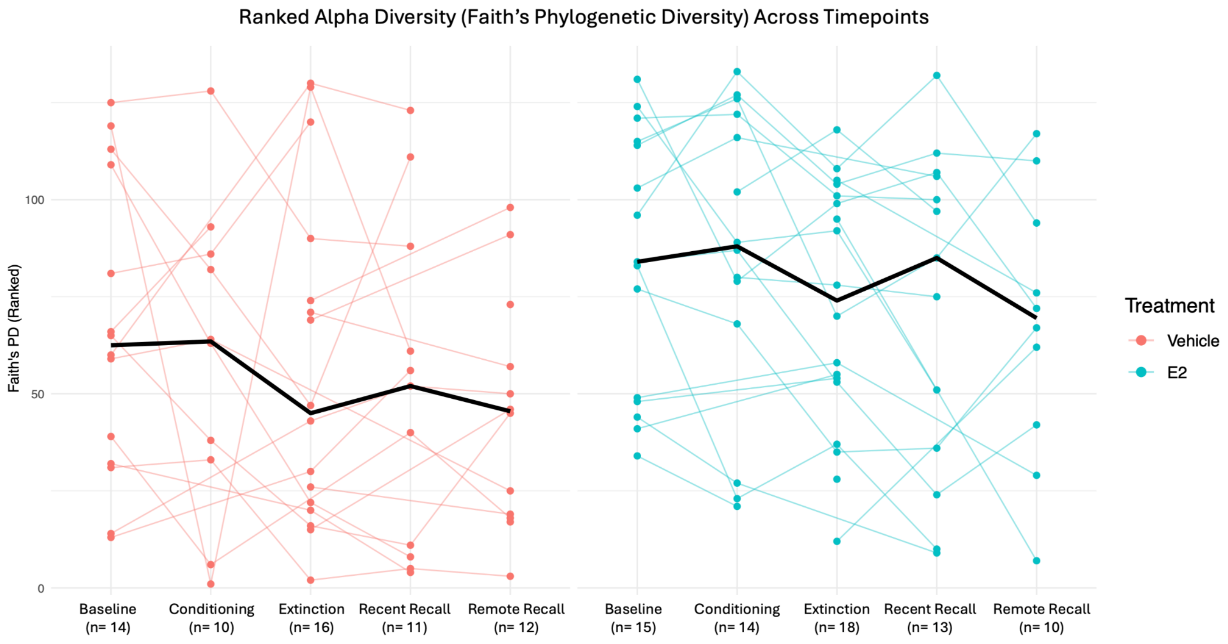

Figure S3. A rank-based linear mixed model analysis revealed no significant fixed effects of treatment or timepoint on ranked alpha diversity (Faith's PD) across all timepoints.

Serum Estradiol Concentration

Timepoint	Treament	Mean (pg/ml)	Standard Error	n
Baseline (Metestrus)	Vehicle	114.88	10.51	7
	E2	97.00	17.06	8
Baseline (Proestrus)	Vehicle	170.74	15.6	6
	E2	182.38	16.89	5
Habituation and Conditioning	Vehicle	117.12	8.55	13
	E2	127.15	15.07	12
Extinction	Vehicle	131.22	17.02	14
	E2	**1824.47 ***	300.66	12
Recent Recall	Vehicle	127.77	9.79	13
	E2	145.82	11.74	14
Remote Recall	Vehicle	162.4	55.1	12
	E2	108.23	26.25	8

Table S1. A linear mixed effects model revealed an effect of time on serum E2 concentration [F (5, 26.767) = 7.320, $p < 0.001$]. Pairwise comparisons demonstrated that serum E2 levels of E2-treated rats at extinction were significantly higher than all other timepoints ($p < 0.05$).

Fixed Effects Table from Rank-Based Linear Mixed Model

	Faith's Phylogenetic Diversity (Faith's PD) Ranked					
**Term**	**Estimate**	**Std. Error**	**CI Lower**	**CI Upper**	**t**	**p**
Intercept	66.000	9.424	47.287	84.714	7.004	**< .001**
Treatment: E2 vs. Vehicle	18.145	13.105	-7.886	44.175	1.385	0.17
Timepoint: Conditioning	-9.733	10.400	-30.407	10.940	-0.936	0.352
Timepoint: Extinction	-11.459	9.467	-30.265	7.347	-1.210	0.229
Timepoint: Recent Recall	-12.163	10.080	-32.201	7.876	-1.207	0.231
Timepoint: Remote Recall	-11.942	11.012	-33.781	9.897	-1.084	0.281
E2 × Conditioning	4.371	14.010	-23.478	32.220	0.312	0.756
E2 × Extinction	-3.203	13.084	-29.196	22.789	-0.245	0.807
E2 × Recent Recall	-9.853	13.946	-37.574	17.867	-0.707	0.482
E2 × Remote Recall	-11.175	15.435	-41.805	19.455	-0.724	0.471
**Random Effects**						
σ^2	603.75					
$\tau_{00\ RatID}$	807.19					
ICC	0.57					
N_{RatID}	42					
Observations	133					
Marginal R^2 / Conditional R^2	0.070 / 0.602					

Table S2. A rank-based linear mixed model was conducted to examine the effects of treatment (vehicle vs. E2) and timepoint (Baseline, Conditioning, Extinction, Recent Recall, and Remote Recall) on Faith's Phylogenetic Diversity (Faith's PD). The model included a random intercept for each subject to account for repeated measures. The analysis revealed no main effects or interaction [$p > 0.05$]. The model accounted for a substantial proportion of variance when including subject-level random effects (conditional $R^2 = 0.602$), though fixed effects alone explained relatively little variance (marginal $R^2 = 0.070$). The intraclass correlation coefficient (ICC = 0.57) indicated a high degree of consistency within individuals across timepoints.

References

1. Chen See JR, Amos D, Wright J, Lamendella R, Santanam N (2021): Synergistic effects of exercise and catalase overexpression on gut microbiome. *J Environ Microbiol* 24: 4220–4235.
2. Sapp PA, Kris-Etherton PM, Petersen KS (2022): Peanuts or an Isocaloric Lower Fat, Higher Carbohydrate Nighttime Snack Have Similar Effects on Fasting Glucose in Adults with Elevated Fasting Glucose Concentrations: a 6-Week Randomized Crossover Trial. *J Nutrition* 152: 153–162.
3. Walters W, Hyde ER, Berg-Lyons D, Ackermann G, Humphrey G, Parada A, *et al* (2015): Improved Bacterial 16S rRNA Gene (V4 and V4-5) and Fungal Internal Transcribed Spacer Marker Gene Primers for Microbial Community Surveys. *mSystems*, 1:, e00009-15. https://doi.org/10.1128/mSystems.00009-15
4. Bolyen E, Rideout JR, Dillon MR, Bokulich NA, Abnet CC, Al-Ghalith GA, *et al* (2019): Reproducible, Interactive, Scalable and Extensible Microbiome Data Science Using QIIME 2. *Nature Biotechnology* 37: 852–57. https://doi.org/10.1038/s41587-019-0209-9.
5. Rognes T, Flouri T, Nichols B, Quince C, Mahé F (2016): VSEARCH: A Versatile Open Source Tool for Metagenomics. *PeerJ* 4. https://doi.org/10.7717/peerj.2584.
6. Callahan BJ, McMurdie PJ, Rosen MJ, Han AW, Johnson AJA, Holmes SP (2016): DADA2: High-Resolution Sample Inference from Illumina Amplicon Data." *Nature Methods* 13: 581–83. https://doi.org/10.1038/nmeth.3869.
7. Faith DP, Baker AM (2007): Phylogenetic Diversity (PD) and Biodiversity Conservation: Some Bioinformatics Challenges. *Evolutionary Bioinformatics Online* 2: 121–28.

8. Pielou EC (1966): The Measurement of Diversity in Different Types of Biological Collections. *J of Theoretical Biology* 13: 131–44. https://doi.org/10.1016/0022-5193(66)90013-0.
9. DeSantis TZ, Hugenholtz P, Larsen N, Rojas M, Brodie EL, Keller K, Huber T, Dalevi D, Hu P, Andersen GL (2006): Greengenes, a Chimera-Checked 16S RRNA Gene Database and Workbench Compatible with ARB. *Applied and Environmental Microbiology* 72: 5069–72. https://doi.org/10.1128/AEM.03006-05.
10. Paulson JN, Stine OC, Bravo HC, Pop M (2013): Robust Methods for Differential Abundance Analysis in Marker Gene Surveys. *Nature Methods* 10: 1200–1202. https://doi.org/10.1038/nmeth.2658.
11. Lozupone CA, Hamady M, Kelley ST, Knight R (2007): Quantitative and Qualitative Beta Diversity Measures Lead to Different Insights into Factors That Structure Microbial Communities. *Applied and Environmental Microbiology* 73: 1576–85. https://doi.org/10.1128/AEM.01996-06.
12. Segata N, Izard J, Waldron L, Gevers D, Miropolsky L, Garrett WS, Huttenhower C (2011): Metagenomic Biomarker Discovery and Explanation. *Genome Biology* 12: R60. https://doi.org/10.1186/gb-2011-12-6-r60.
13. Espinosa E, Curtis KS (2018): Increased locomotor activity in estrogen-treated ovariectomized rats is associated with nucleus accumbens dopamine and is not reduced by dietary sodium deprivation. *Integrative Zoology*, 13: 783–794. https://doi.org/10.1111/1749-4877.12333

14. Krentzel AA, Proaño S, Patisaul HB, Meitzen J (2020): Temporal and bidirectional influences of estradiol on voluntary wheel running in adult female and male rats. *Hormones and Behavior*, 120: 104694. https://doi.org/10.1016/j.yhbeh.2020.104694

15. Gruene TM, Flick K, Stefano A, Shea SD, Shansky RM (2015): Sexually divergent expression of active and passive conditioned fear responses in rats. *eLife*, 4: e11352. https://doi.org/10.7554/eLife.11352
